# Supplementary material for: Identification of photoperiod-induced specific miRNAs in the adrenal glands of Sunite sheep (Ovis aries)
Source: Front Vet Sci. 2022 Jul 22;9:888207. doi: 10.3389/fvets.2022.888207 (PMC9354845; doi:10.3389/fvets.2022.888207)
Supplement: Supplementary file 1 [file Data_Sheet_1.ZIP › Supplementary file/miRDeep2 file.zip!/miRDeep2 file/Know_Structure/SP42A1/oar-mir-218a.pdf]

miRBase precursor : oar-mir-218a
Total read count : 23029
oar-miR-218a read count : 23028
remaining reads : 1

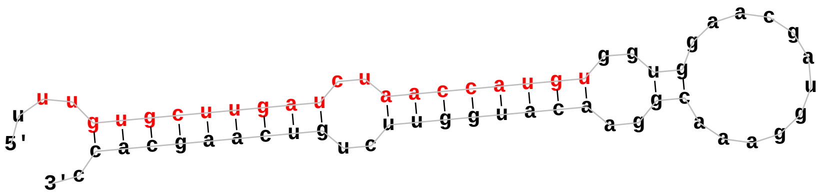

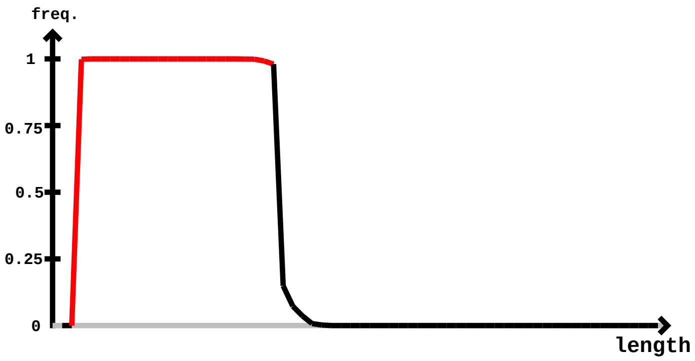


```
      oar-miR-218a
5'- uuugugcuugaucuaaccaugugguggaacgauggaaacggaacaugguucugucaagcacc   -3'    exp
    ...(((((((((..((((((((..((............))..))))))))..))))))))).    reads      mm          sample
    uuugugcuugaucuaaccaugu........................................    1          0           seq
    .uugugcuugaucuaacc............................................    12         0           seq
    .uugugcuugaucuaacA............................................    1          1           seq
    .uugugcuugaucuaacca...........................................    12         0           seq
    .uugugcuugaucuaaccau..........................................    150        0           seq
    .uugugcuugaucuaacAaug.........................................    1          1           seq
    .uugugcuugaucuaaccaug.........................................    277        0           seq
    .uugugcuugaucuaacAaugu........................................    33         1           seq
    .uugugcuugaucuaaccaugu........................................    19100      0           seq
    .uugugcuugaucuaaccaugug.......................................    1757       0           seq
    .uugugcuugaucuaaccaugugg......................................    824        0           seq
    .uugugcuugaucuaaccauguggu.....................................    658        0           seq
    .uugugcuugaucuaaccauguggU.....................................    116        1           seq
    .uugugcuugaucuaaccauguggug....................................    3          0           seq
    .uugugcuugaucuaaccauguggUg....................................    48         1           seq
    .uugugcuugaucuaaccauguggugggaac...............................    1          0           seq
    ..ugugcuugaucuaaccaugu........................................    11         0           seq
    ..ugugcuugaucuaaccaugug.......................................    2          0           seq
    ..ugugcuugaucuaaccaugugg......................................    11         0           seq
    ..ugugcuugaucuaaccauguggu.....................................    6          0           seq
    ..ugugcuugaucuaaccauguggU.....................................    3          1           seq
    ..ugugcuugaucuaaccauguggUg....................................    1          1           seq
    ....ugcuugaucuaaccauguggU.....................................    1          1           seq
```
